# Does Symptom Recognition Improve Self-Care in Patients with Heart Failure? A Pilot Study Randomised Controlled Trial

**DOI:** 10.3390/nursrep11020040

**Published:** 2021-06-01

**Authors:** Joana Pereira Sousa, Hugo Neves, Miguel Pais-Vieira

**Affiliations:** 1 Institute of Health Sciences, Universidade Católica Portuguesa, 4169-005 Porto, Portugal; 2 School of Health Sciences, Polytechnic of Leiria, 2411-901 Leiria, Portugal; 3 Health Sciences Research Unit: Nursing—UICISA:E, Nursing School of Coimbra (ESEnfC), 3000-232 Coimbra, Portugal; hugoneves@esenfc.pt; 4 Center for Innovative Care and Health Technology—CiTechCare, 2411-901 Leiria, Portugal; 5 Institute of Biomedicine—iBiMED, Department of Medical Sciences, University of Aveiro, 3810-193 Aveiro, Portugal; miguelpaisvieira@ua.pt

**Keywords:** heart failure, self-care behaviours, symptom recognition, nurse-led programme

## Abstract

Patients with heart failure have difficulty in self-care management, as daily monitoring and recognition of symptoms do not readily trigger an action to avoid hospital admissions. The purpose of this study was to understand the impact of a nurse-led complex intervention on symptom recognition and fluid restriction. A latent growth model was designed to estimate the longitudinal effect of a nursing-led complex intervention on self-care management and quality-of-life changes in patients with heart failure and assessed by a pilot study performed on sixty-three patients (33 control, 30 intervention). Patients in the control group had a higher risk of hospitalisation (IRR 11.36; *p* < 0.001) and emergency admission (IRR 4.24; *p* < 0.001) at three-months follow-up. Analysis of the time scores demonstrated that the intervention group had a clear improvement in self-care behaviours (βSlope. Assignment_group = −0.881; *p* < 0.001) and in the quality of life (βSlope. Assignment_group = 1.739; *p* < 0.001). This study supports that a nurse-led programme on symptom recognition and fluid restriction can positively impact self-care behaviours and quality of life in patients with heart failure. This randomised controlled trial was retrospectively registered (NCT04892004).

## 1. Introduction

Symptom perception is associated with self-care management, within the middle-range theory of self-care for chronic illness defined by Riegel, Jaarsma, and Strömberg [1]. In this paradigm, symptom perception includes symptom monitoring and recognition and is a challenging task to be undertaken by patients with heart failure (HF). Experiencing a symptom consists of awareness by a person of the bodily changes and how they can affect him/her [2]. It means that a person has to detect the symptom in a timely fashion, evaluate its meaning, and finally, take action in response to symptom perception [3,4,5]. Being aware of body changes related to heart failure escalation symptoms requires patients to have enough information about it. Previous studies suggest that this is frequently not the case [6,7], as most patients might have chronic conditions, which interfere with HF symptoms [7]. Furthermore, patients who experience an acute onset of symptoms typically seek help earlier than those who experience a gradual worsening of symptoms, who only seek help when symptoms are severe [8].

Symptom recognition, therefore, seems to be a barrier to healthy self-care because patients fail to understand the signs and symptoms of heart failure after hospital discharge for heart failure exacerbation [6]. At home, patients have to implement effective self-care techniques for daily activities by themselves, and finding the balance may be harder without the help of healthcare professionals [6]. The most frequent HF symptoms reported by patients are dyspnoea, weight gain, wake disturbance, and oedema [6,9]. However, the association between two or more symptoms, misunderstanding HF symptoms from other chronic diseases, and daily symptom fluctuations may lead patients to identify symptoms as normal, resulting in a stay at home, waiting for symptoms to disappear [6,10].

Heart failure disease management programmes (HF-DMPs) allow a structured follow-up approach where patients receive education on major topics of HF and where medical treatment and psychological support optimise the patients’ disease condition [11,12,13]. HF patients are managed as a whole in concordance with the European Society of Cardiology (ESC) [14]. Besides HF-DMPs, nurse-led education programmes target disease knowledge and self-care behaviours and have also demonstrated good disease management by HF patients [15,16]. These programmes enable patients to make conscious choices and decisions about their health status.

A pilot study centred on developing an intervention on symptom management and its inclusion in a nurse-led programme was designed to improve self-care behaviours and decrease hospitalisations of HF patients. Self-care is complex, and structuring an educational area, for example, on fluid restriction, may help HF patients better control their health condition in a balanced way.

The aims of this study were as follows: (a) understand the longitudinal impact of a nurse-led programme based on intervention on fluid management on HF patients’ self-care management and (b) analyse the quality of life (QoL) changes during the nurse-led programme.

## 2. Materials and Methods

In this pilot study, 63 patients (47 males and 16 females) in New York Heart Association (NYHA) functional class II–III, according to their medical record at admission, were recruited in a hospital setting after discharge from a heart failure unit. Inclusion criteria were adults aged >18 years old, diagnosed with HF, and with no cognitive disability. Exclusion criteria included patients placed on the heart transplant waiting list and patients in class IV NYHA.

**Figure 1:**
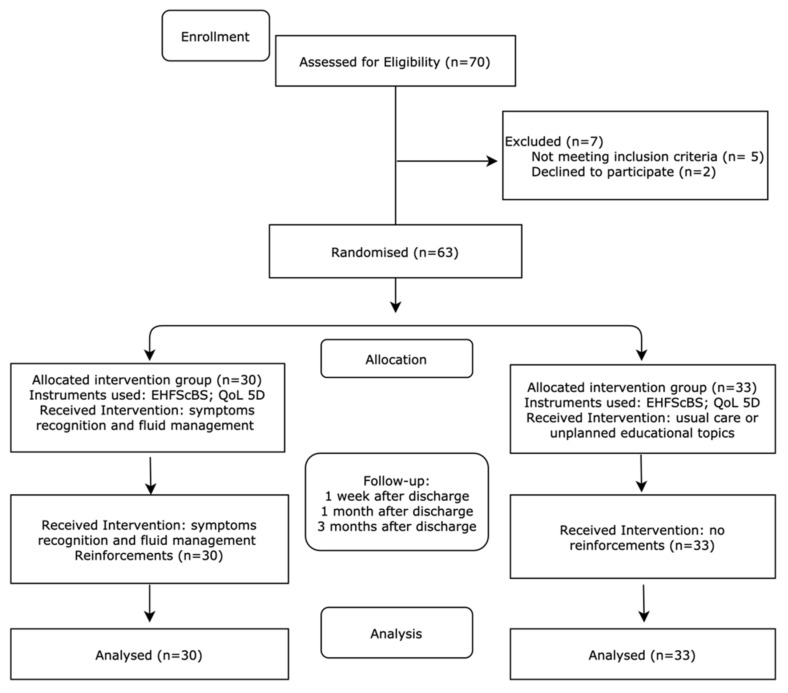
Pilot study RCT CONSORT flowchart.

Patients were allocated into a control group (CG, *n* = 33) or an intervention group (IG, *n* = 30) through the computerised random allocation generator at http://random.org (accessed on 3 September 2014). When a patient was admitted into the hospital, and after doctor’s registries at the patient’s clinical file, the leading investigator was contacted, who then contacted the patient if they met inclusion criteria and ran the computerised random allocation generator. Patients allocated to the CG got odd numbers, and patients to the IG got even numbers. In the CG, patients receive usual care. Patients were recruited during hospital admission to a Heart Failure Unit in a central hospital from a southern European country. The pilot study was performed for three months per patient, with four moments of assessment (baseline, first-week follow-up, first-month follow-up, and third-month follow-up), starting in September of 2014 and ending in December of 2017, as depicted in Figure 1. This timeline is due to the leading investigator being a full-time nurse whose investigation period is apart from her job.

This study protocol was registered retrospectively as a randomised controlled trial (RCT; with the number NCT04892004, at ClinicalTrials.gov).

**Figure 2:**
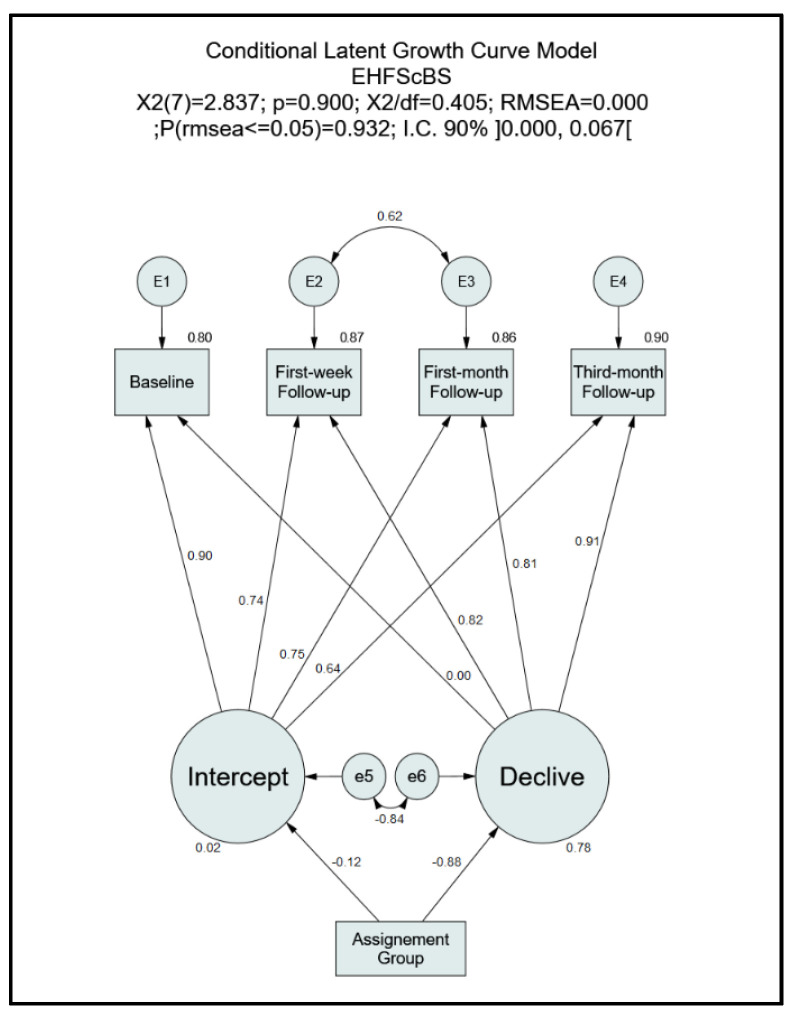
Conditional Latent Growth Curve Model—EHFScBS (European Heart Failure Self-care Behaviour Scale).

**Figure 3:**
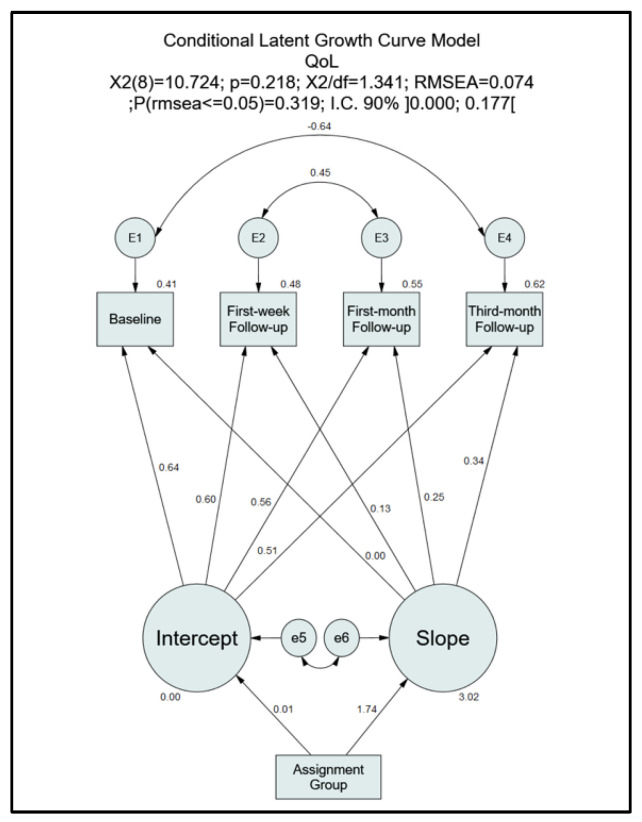
Conditional Latent Growth Curve Model—QoL (Quality of Life).

In order to understand the intervention’s impact on self-care behaviours and quality of life, classical statistical tests (independent samples *t*-test, Pearson’s correlation, chi-squared test, Fisher’s exact test, U Mann–Whitney) for each moment of assessment were carried out using IBM SPSS software v.24.0 (IBM Corp., Armonk, NY, USA). Prerequisites for testing were carried out before the selection of the appropriate statistical test. As for the assessment of the impact of the intervention at all moments on self-care behaviours (Figure 2) and quality of life (Figure 3), two theoretical Conditional Latent Growth Curve Models [17] were tested through the use of the software IBM SPSS Amos for structural equation modelling v.24.0 (IBM Corp., Armonk, NY, USA). No regression weight was predefined between the assignment group and the intercept and slope. Minimum regression weight (0) and maximum regression weight (1) were applied between slope and the baseline and third-month follow-up, respectively, with no predefined regression weight in the remaining follow-up moments. A predefined regression weight of 1 was applied between the intercept and all the follow-up moments. A *p*-value of 0.05 was considered for testing. Effect size and confidence intervals were also calculated.

The study was approved by the Ethics Committee of the Hospital (CHUC-032-14, approved in May 2014), with individual informed consent obtained before inclusion in the study, following the principles outlined in the Declaration of Helsinki [18] and the Declaration of Taipei [19]. Researchers had access to anonymised material, and this guaranteed the confidentiality of institutions. All rights regarding data protection were respected.

### 2.1. Intervention Design

The intervention developed for this pilot study emerged from the aggregation of qualitative research [20] and systematic literature review data, according to the complex intervention method by Medical Research Council (MRC) [21]. The goal of the intervention is to improve HF patients’ ability to recognise symptom escalation, which, as discussed above, leads to clinical deterioration. However, although the ESC guidelines describe educational topics [14] to include in HF-DMPs, the present study intended to focus on the educational topic that patients could more easily manage. These educational topics emerged from the results of previous qualitative research, where HF patients considered a fluid overload and symptom recognition as the most difficult to detect and manage by patients in this setting [20], and the synthesis of the evidence available in the topic.

At first contact, the patient in the IG received a leaflet, which included information about HF, primary symptoms, awareness of its detection, and the fluid management plan. They were also given a weight diary, which helped them recall weight fluctuation and contact the nurse or doctor to call for help at an early stage and avoid hospitalisation. Patients had to explain on follow-up contacts what they understood by HF, which are the main symptoms, whether they were experiencing any of them, and which difficulties they faced in managing fluid restriction and weight control. The leading investigator validated the information and retaught contents if required. Therefore, when patients indicated difficulty in processing information at once or found it too complex, the contents were repeated using a different choice of words.

A nurse with expertise in HF provided this intervention to participants in the IG and addressed reinforcements on (a) an explanation of signs and symptoms of HF and how to recognise them; (b) the importance of daily fluid management, by planning an intake of 1.5–2 L of liquids per day (e.g., soup, milk, coffee, water, tea, and yoghurt); and (c) when doctors or nurses should be contacted (when symptom escalation or a weight gain of 2 kg in three days or 5 kg in a week were detected, according to European Society of Cardiology heart failure guidelines [14]).

As the intervention could negatively impact the stress associated with the perception of symptoms with an impact on the quality of life, and as the standard Portuguese follow-up of these patients was inexistent, the CG only received information at baseline. Standard information is defined as the routine, unplanned care provided for HF patients and not personalised as in the case of the intervention administered to the IG.

### 2.2. Instruments

Self-care behaviours were measured using the 12-item European Heart Failure Self-care Behaviour Scale (EHFScBS), with a Cronbach’s α of 0.85 [22] at the time of development. Quality of life (QoL) was measured using the EuroQol-5D (EQ-5D), with a Cronbach’s α of 0.716 [23] at the time of development. On the EHFScBS, lower scores report better self-care behaviours. All participants (CG and IG) filled the instrument at all moments of contact: at the time of study admission, one week after discharge follow-up, one month after discharge follow-up, and three months after discharge follow-up.

## 3. Results

Sample characteristics show a higher percentage of men (74.60%) living with a companion (69.84%), with no significant difference between the IG and CG (range: 73.33–75.76; *p* = 0.825; *d* = 0.056). The mean age was 54.83 years (10.28), with no significant difference (*p* = 0.630; *d* = 0.122) between the CG [54.42 (10.54)] and the IG [55.27 (10.15)]. Most individuals had an NYHA functional class of III (73.02%) with no significant difference between the IG and CG (range: 63.33–81.81; *p* = 0.099; *d* = 0.425), as presented in Table 1.

### 3.1. Symptom Recognition

The IG patients demonstrated a positive, progressive evolution of knowledge and understanding of HF, displaying an improvement of disease understanding in all follow-up moments (*p* < 0.05; Φ > 0.5). In other words, when questioned were posed about which signs and symptoms were relevant or when asked to describe which actions to take in a given circumstance related to HF (e.g., when to go to the hospital), a more significant fraction of IG patients answered correctly. On symptom recognition, IG patients could accurately identify HF symptoms (*p* < 0.05), with the presence of a very high effect size on the symptoms “Daily weight record”, “Sudden weight gain”, and “Fluid restriction accomplishment” (Φ > 0.5), and a high effect size on the other HF symptoms (0.25 < Φ ≤ 0.5), in Table 2. Only the topic of sleeping seated or with pillows did not evidence an association with the CG or IG (*p* > 0.05).

### 3.2. Emergency and Hospital Admissions

In the CG, HF patients went to the emergency department more often than those in the IG, at one month (*p* = 0.014; 95% CI 1.10–60.62) and three months follow-up (*p* < 0.05; 95% CI 2.94–43.96), with an 8.18 higher risk at the end of the first month of discharge, and 11.36 higher risk at the three months follow-up. Regarding hospital admission, at one-month follow-up, patients in the CG had a 3.64 higher risk of hospital admission, but no significant association between these variables was found (*p* = 0.357; 95% CI 0.43–30.45). However, at three months follow-up, patients in the CG had a 4.24 higher risk of being admitted into hospital (*p* < 0.05; 95% CI 2.04–8.80), as presented in Table 3.

### 3.3. Self-Care Behaviours

As the initial theoretical model did not present a good fit, analysis of the modification indices (MI > 4, *p* < 0.05) and respective correlation of measurement errors demonstrated the need to remove the linear growth coefficients between the “first-week follow-up” and the “first-month follow-up”. The global significance of the conditioning variables was assessed through the chi-square differences between the model with a fixed effect of 0 and the model with random effects for the variable “Assignment Group” (ΔX^2^(2) = 115.57; *p* < 0.001), with this variable being significant in the adjusted conditioned growth curve model.

Analysis of the final theoretical model (Figure 2) evidenced the presence of an excellent model fit to the variance structure, covariance, and means of the sample under study (X^2^(7) = 2.8; *p* = 0.900; X^2^/df = 0.405; RMSEA < 0.001 and P [rmsea ≤ 0.05] = 0.932; RMSEA IC 90% ]0.000; 0.067[). There was no evidence of the “Assignment Group” effect on the intercept (βIntercept. Assignment_group = −0.125; *p* = 0.375). Regarding the slope, there was a significant negative effect of the intervention (βSlope. Assignment_group = −0.881; *p* < 0.001) on the self-care behaviour scores, indicating that the intervention positively influenced a progressive improvement in these behaviours. There was significant growth in self-care behaviours at the first-week follow-up (M(%) = 0.785; SE = 0.053; Z = 14.8; *p* < 0.001), representing 78.5% of the total growth. Despite the significant increase in self-care behaviours at the first-month follow-up (M(%) = 0.762; SE = 0.053; Z = 14.4; *p* < 0.001) when compared to the basal scores, with 76.2% of the total growth represented at this moment, the model suggested a slight stabilisation from the first-week follow-up to the first-month follow-up. The estimation of both parameters presented significant variances (V(Intercept) = 94.08; SE = 21.24; *p* < 0.001 and V(Slope) = 43.04; SE = 16.71; *p* = 0.010), indicating inter-variability in the basal scores of self-care behaviours and the growth rates. The mean basal score of self-care behaviours was 40.00 (SE = 1.90; *p* < 0.001), while the mean growth rate was 5.11 (SE = 1.62; *p* = 0.002).

### 3.4. Quality of Life

The initial theoretical model required the analysis of the modification indices (MI > 4, *p* < 0.05), as there was a violation of the fit indices. Through analysis of the correlation of measurement errors, we proceeded with the removal of the linear growth coefficients between the “baseline” and the “third-month follow-up” and between the “first-week follow-up” and the “first-month follow-up”. The variable “Assignment Group” was significant in the adjusted conditioned growth curve model (ΔX^2^(2) = 35.05; *p* < 0.001).

Except for the RMSEA 90% CI upper limit (>0.10), all the fit indices evidenced the presence of an almost perfect model fit to the variance structure, covariance, and means of the sample under study (X^2^(8) = 10.7; *p* = 0.218; X^2^/df = 1.341; RMSEA = 0.074 and P [rmsea ≤ 0.05] = 0.319; RMSEA IC 90% ]0.000; 0.177[) of the final theoretical model (Figure 3). There was no evidence of the “Assignment Group” effect on the intercept (βIntercept. Assignment_group = −0.008; *p* = 0.966). Regarding the slope, there was a significant positive effect of the intervention (βSlope. Assignment_group = 1.739; *p* < 0.001) on the quality-of-life scores, suggesting that the intervention positively influenced a progressive improvement in this variable. Overall growth was stable throughout the various moments of assessment (*p* > 0.05). The model also suggested inter-variability in the basal scores of QoL (V(Intercept) = 65.93; SE = 27.40; *p* = 0.013) but not in the growth rates (V(Slope) = −6.04; SE = 6.11; *p* = 0.261). The mean basal score of QoL was 68.76 (SE = 2.10; *p* < 0.001), while the mean growth rate was −2.49 (SE = 0.79; *p* = 0.002).

### 3.5. Self-Care Behaviours and Quality of Life

No statistically significant relation was found between self-care behaviours and QoL (*p* > 0.05) (Table 4). Only in the three-month follow-up of the IG HF patients, a significant relation between self-care behaviours and QoL (ρ < 0; *p* < 0.05) was found, with lower scores of EHFScBS (better self-care behaviours) relating to higher scores of EQ-5D (higher quality of life) (Table 5).

## 4. Discussion

In this pilot study, an intervention on symptom recognition by HF patients was tested. A latent growth model showed that patients in the intervention group had improved self-care behaviours. They could better interpret and recognise early signs and symptoms of HF decompensation, with better outcomes regarding the number of visits to the emergency room and hospital readmissions. Patients in the intervention group also had better scores in quality of life at three-month study time.

HF patients from both groups had a variation of weight decrease between hospital admission and discharge, reporting a clinical deterioration. Throughout the study, when asked about symptoms of HF, “weight increase” was primarily interpreted by patients in the IG. Still, symptom interpretation had a slight decrease at three-months follow-up. It may suggest that weight gains linked to oedema are not interpreted as severe, possibly because congestion has a slow onset and does not immediately trigger patients’ symptom perception of disease decompensation [4,20,24]. An alternative explanation for this increase in symptoms could be that managing multiple topics may become difficult for these patients in the long term. Usually, patients who experience more severe symptoms are more engaged in self-care [9]. This gap between symptom recognition and taking action to promote health leads to a delay in care-seeking [8,10,25].

### 4.1. Hospital and ER Admission

Patients in both groups were admitted into the ER. However, patients in the IG reported fewer ER visits than patients in the CG, at one month and three-month follow-up. Those who were admitted to the hospital had a considerable difference at three-months follow-up between groups. Likewise, the literature indicates longer interventions decrease the mortality rate for each month of intervention, reduce hospitalisation risk, and decrease hospital admission at the end of six months [26]. When HF patients are included in disease management programmes, hospital readmissions are lower because of improvement in the patients’ knowledge and better self-care behaviours [15,27,28]. Our results support these findings, as patients are more engaged in self-care behaviours and more committed to improving their health status while avoiding hospitalisations and ER visits [27].

### 4.2. Self-Care Behaviour Improvement

Patients in the IG had a more significant improvement in self-care, measured by EHFScBS. At baseline, both patients at the CG and the IG had similar scores for self-care behaviours. However, during the three-month study period, combined with reinforcements at all moments in the IG, there was a progressive and robust improvement in explaining what HF is. The intervention also improved in identifying most HF symptoms, especially “Weight diary”, “Sudden weight gain”, and “Fulfil with water restriction”. These results indicate that participants in the IG could understand HF symptoms and be competent in naming them, improving their awareness of symptom recognition. Symptom interpretation and recognition are hard to implement, and patients who are not aware of them are more prone to demonstrate poor self-care [6]. In contrast, those who experience more severe symptoms are more engaged in self-care [9,29], mostly recalling prior experiences.

When symptom interpretation and recognition are integrated into a nurse-led programme with education reinforcements at all follow-up moments, HF patients significantly improve their self-care [15]. In our study, patients interacted with the self-care information through various means such as leaflets, explaining concepts related to the disease management to their nurse, and using a weight diary. These patients informally reported them to be generally helpful and easy to understand. These results support previous studies where educational programmes provided by nurses for HF patients have increased their knowledge about the disease and self-care behaviours after a week, a month, and even after three months [15,30]. In our study, this improvement was clear between baseline and the first-week follow-up, and between the first-month and the third-month follow-ups, with stabilisation in self-care behaviour improvement between first-week and first-month follow-ups.

Managing self-care depends on how a person understands the advantage of clinical stability and avoiding hospital admissions. Patients need to be informed of their health status, what symptoms and signs to recognise, and who to contact if doubts occur. In this context, using tailored education, healthcare professionals may assign relevant information for each patient at the right time in the right moment. Moreover, it can be performed either at the clinic by telehealth appointments or through telephone follow-ups. In this case, one possible interpretation of these findings is that patients were more engaged in the learning process until the first week of the study and, therefore, were careful in monitoring daily symptoms (which stabilised), in line with the results from the first-month follow-up. Educational reinforcements, different for each patient due to their different needs of apprenticeship, were crucial to make patients aware of the need to keep monitoring symptoms and, therefore, engage in self-care behaviours.

### 4.3. Quality of Life

The interventional programme designed for this study supports a positive influence on QoL for HF patients. Other studies have reported that nursing educational programmes evaluating fatigue levels, self-care education, and training for 12 weeks were associated with improvements in fatigue and QoL [31]. Nevertheless, shorter studies reported QoL improvement in HF patients admitted into a multidisciplinary transition-to-care program [32].

The findings from the present study are in line with the conclusions of previous studies, where disease management programmes or nurse-led programmes have improved HF patients’ quality of life, therapeutic regimen adherence, and better self-care behaviours.

HF is a chronic condition that patients must learn how to live with. Disease management programmes, which include many topics for educating people about the disease, are intended to help patients manage their health–disease status to change self-care behaviours. Focusing on symptom-recognition education as an essential topic to enable HF patients to engage in better self-care and quality of life.

### 4.4. Limitations

This was a pilot study, and therefore the main limitation to the interpretation of the present results is the sample size. In addition, the present study did not precisely control for the possibility of patients finding the information given in the intervention to be too complex or confusing. Therefore, the current results may be, in part, affected by this. Another limitation relates to the present RCT being registered retrospectively due to poor study design, which introduced a bias in the study. As discussed above, our results align with previous reports on the effects of nurse-led HF management programmes. We will repeat the present study with larger samples, multicentred research, and a liaison between the hospital and community care for centred patient management in future studies. Furthermore, it will be essential to study the effects of extending follow-up to 12, 18, and 24 months, determining whether the present intervention should be maintained or eventually adapted to these specific periods.

To support the strength of the intervention, fidelity has to be tested in a larger and longer study. Lastly, although we focused our intervention on a small number of variables, previously identified as the most likely to be relevant for self-care in HF patients [33,34,35], other parameters may also be good predictors of intervention and/or disease outcomes. For example, the number of days of in-hospital stays and the weight variation might be accounted to analyse how long patients stabilise their clinical condition and to compare whether the intervention interferes with clinical improvement.

## 5. Conclusions

Disease management programmes include several educational topics. Managing all those topics may be complex for HF patients as they may misunderstand all the information, leading to difficulty in managing self-care. Information therapy seems to be a reliable tool to equip a patient for their self-management. It involves tailored and individualised education, based on evidence, at the right time at the right moment, for each patient. Focusing on symptom recognition and fluid restriction, with individual reinforcements, seems to help HF patients improve and engage in self-care, as information is systematically recalled.

In future studies, mobile apps implementation can be a helper for symptom recognition through programmes that use artificial intelligence to recall signs and symptoms, help the interpretation of those, and contact healthcare professionals, if required.

## Figures and Tables

**Table 1:** Participants’ characteristics.

Characteristics	Total (*n* = 63)	CG (*n* = 33)	IG (*n* = 30)	Statistic	*p*-Value	Effect-Size
Gender, men (%)	74.6	75.76	73.33	0.049 ^a^	0.825	0.056 ^d^
Male (*n*)	47	25	22			
Female (*n*)	16	8	8			
Age, mean (SD)	54.83 (10.28)	54.42 (10.54)	55.27 (10.15)	460.0 ^b^	0.63	0.122 ^e^
Marital status, with companion (%)	69.84	69.69	70	0.001 ^a^	0.979	0.008 ^d^
NYHA class, III class (%)	73.02	81.81	63.33	2.725 ^a^	0.099	0.425 ^d^
Initial weight, mean (SD)	78.73 (15.04)	81.00 (15.56)	76.23 (14.28)	1.261 ^c^	0.212	0.318 ^e^
EHFScBS, mean (SD)	38.81 (10.91)	39.94 (8.27)	37.57 (13.26)	454.0 ^b^	0.572	0.143 ^e^
QoL, mean (SD)	67.86 (12.94)	68.18 (11.78)	67.50 (14.31)	0.207 ^c^	0.837	0.052 ^e^

^a^ Χ^2^—Pearson’s chi-squared test; ^b^ U—Mann–Whitney’s test; ^c^ t—Student’s *t* test; ^d^ Φ—phi; ^e^ *d*—Cohen’s *d.* CG—control group; IG—intervention group; NYHA—New York Heart Association; EHFScBS—European Heart Failure Self-care Behaviour Scale; QoL—quality of life.

**Table 2:** Symptom recognition by HF patients.

	CG	IG	Χ^2^	*p*-Value	Φ
Symptoms and Signs Recognition by Patients	(*n* = 33)	(*n* = 30)			
First-week follow-up					
Weight increase (>2 kg/3 days or 5 kg/week), yes (%)	6.06	36.67	8.988	0.003	0.378
Oedema, yes (%)	48.48	30	2.243	0.134	−0.189
Shortness of breath, yes (%)	48.48	63.33	1.403	0.236	0.149
Fatigue after small effort, yes (%)	45.45	53.33	0.39	0.532	0.079
Sleep seated or with pillows, yes (%)	3.03	16.67	3.391	0.094 ^a^	0.232
Daily weight record, yes (%)	3.03	80	38.895	0	0.786
Recognises rapid increase of weight, yes (%)	0	46.67	19.8	0	0.561
Fulfils fluid restriction, yes (%)	15.15	96.67	42.032	0	0.817
Calls doctor or nurse when detects symptoms early, to avoid hospitalisation, yes (%)	0	6.67	2.272 ^b^	0.223 ^a^	0.19
First-month follow-up					
Weight increase (>2 kg/3 days or 5 kg/week), yes (%)	6.06	33.33	7.58	0.006	0.347
Oedema, yes (%)	45.45	33.33	0.965	0.326	−0.124
Shortness of breath, yes (%)	54.54	76.67	3.384	0.066	0.232
Fatigue after small effort, yes (%)	48.48	60	0.839	0.36	0.115
Sleeps seated or with pillows, yes (%)	0	10	3.465	0.102 ^a^	0.235
Daily weight record, yes (%)	0	56.67	25.611	0	0.638
Recognises rapid increase of weight, yes (%)	0	46.67	19.8	0	0.561
Fulfils fluid restriction, yes (%)	6.06	83.33	38.314	0.000	0.78
Calls doctor or nurse when detects symptoms early, to avoid hospitalisation, yes (%)	3.03	20	4.582	0.047 ^a^	0.27
Third-month follow-up					
Weight increase (>2 kg/3 days or 5 kg/week), yes (%)	0	30	11.55	0.001 ^a^	0.428
Oedema, yes (%)	51.51	86.67	8.961	0.003	0.377
Shortness of breath, yes (%)	48.48	86.67	10.309	0.001	0.405
Fatigue after small effort, yes (%)	54.55	86.67	7.698	0.006	0.35
Sleeps seated or with pillows, yes (%)	3.03	10	1.284	0.340 ^a^	0.143
Daily weight record, yes (%)	0	73.33	37.185	<0.001	0.768
Recognises rapid increase of weight, yes (%)	0	60	27.72	<0.001	0.663
Fulfils fluid restriction, yes (%)	6.06	90	44.569	<0.001	0.841
Calls doctor or nurse when detects symptoms early, to avoid hospitalisation, yes (%)	15.15	40	4.925	0.026	0.28

Χ^2^—Pearson’s chi-squared test; Φ—phi; ^a^ Fisher’s exact test *p*-value; ^b^ Fisher’s exact test.

**Table 3:** ER and hospital admission during study time.

ER and Hospital Admissions	Total (*n* = 63)	CG (*n* = 33)	IG (*n* = 30)	Statistic	*p*-Value	RR (95% CI) *
First-month follow-up						
Resort to the emergency room since last consult, yes (%)	15.87	27.27	3.33	6.744 ^b^	0.014	8.18 (1.10, 60.82)
Hospitalisation, yes (%)	7.94	12.12	3.33	1.661 ^b^	0.357	3.64 (0.43, 30.45)
Three-months follow-up						
Resort to the emergency room since last consult, yes (%)	42.86	75.76	6.67	30.630 ^b^	<0.001	11.36 (2.94, 43.96)
Hospitalisation, yes (%)	53.97	84.85	20.00	26.601 ^a^	<0.001	4.24 (2.04, 8.80)

* For control group ^a^ Χ^2^—Pearson’s chi-squared test; ^b^ Fisher’s exact test.

**Table 4:** Correlation between CG’s QoL and EHFScBS.

		EHFScBS Baseline	EHFScBS 1 Week	EHFScBS 1 Month	EHFScBS 3 Months
QoL baseline	Pearson Correlation	0.066	0.094	0.022	0.218
	*p*-value (2-tailed)	0.714	0.603	0.905	0.223
	*n*	33	33	33	33
QoL 1 week	Pearson Correlation	0.024	−0.035	0.029	0.058
	*p*-value (2-tailed)	0.896	0.848	0.875	0.749
	*n*	33	33	33	33
QoL 1 month	Pearson Correlation	0.050	0.063	0.049	0.090
	*p*-value (2-tailed)	0.783	0.726	0.789	0.620
	*n*	33	33	33	33
QoL 3 months	Pearson Correlation	0.068	−0.046	−0.032	0.008
	*p*-value (2-tailed)	0.707	0.798	0.859	0.965
	*n*	33	33	33	33

**Table 5:** Correlation between IG’s QoL and EHFScBS.

		EHFScBS Baseline	EHFScBS 1 Week	EHFScBS 1 Month	EHFScBS 3 Months
QoL baseline	Pearson Correlation	0.186	0.229	0.132	0.124
	*p*-value (2-tailed)	0.326	0.224	0.487	0.514
	*n*	30	30	30	30
QoL 1 week	Pearson Correlation	0.030	−0.171	−0.138	−0.145
	*p*-value (2-tailed)	0.874	0.365	0.467	0.445
	*n*	30	30	30	30
QoL 1 month	Pearson Correlation	0.104	0.020	−0.085	0.025
	*p*-value (2-tailed)	0.586	0.916	0.654	0.894
	*n*	30	30	30	30
QoL 3 months	Pearson Correlation	0.041	0.329	0.242	−0.375 *
	*p*-value (2-tailed)	0.831	0.076	0.197	0.041
	*n*	30	30	30	30

* Correlation is significant at the 0.05 level (2-tailed).

## Data Availability

This data can be found here: [https://repositorio.ucp.pt/bitstream/10400.14/32147/1/JoanaPereiraSousa_Tese.pdf].
